# Metal-doped silicate and phosphate glasses for antibacterial dental biomaterials

**DOI:** 10.1080/26415275.2023.2284372

**Published:** 2023-12-04

**Authors:** Haruaki Kitagawa, Tomoki Kohno, Fan Deng, Gabriela L Abe, Hirohiko Sakai, Yo-Shiuan Fan, Tingyi Wu, Jun-ichi Sasaki, Satoshi Imazato

**Affiliations:** a Department of Dental Biomaterials, Osaka University Graduate School of Dentistry, 1-8 Yamadaoka, Suita, Osaka, Japan; b Joint Research Laboratory of Advanced Functional Materials Science, Osaka University Graduate School of Dentistry, 1-8 Yamadaoka, Suita, Osaka, Japan

**Keywords:** Metal-doped glass, silicate glass, phosphate glass, antibacterial activity, dental biomaterials

## Abstract

Owing to the development of glass 45S5 (Bioglass®) comprising 45 mol% SiO_2_, 24.5 mol% Na_2_O, 24.5 mol% CaO, and 6 mol% P_2_O_5_, different compositions of silicate glasses have been developed. When these silicate glasses contact an aqueous environment, such as body fluids, they induce apatite layer formation on their surfaces owing to ion exchange. In addition to promoting hard tissue formation, researchers have sought to enhance the antibacterial properties of these glasses, thereby resulting in the development of metal-doped silicate glasses. The addition of antibacterial metals (silver, copper, zinc, and gallium) to silicate glass offers a promising avenue for combating oral pathogens. In recent years, there has been growing interest in metal-doped phosphate glasses. The release of metal ions can be regulated by modifying the dissolution rate of the phosphate glasses. This review summarizes the metal-doped silicate and phosphate glasses that confer antibacterial activity. Future strategies for the development of dental biomaterials that incorporate metal-doped glass and exhibit antibacterial effects are discussed.

## 1. Introduction

An ion-releasing glass is a type of glass that can release certain ions and stimulate desirable biological responses. In the 1960s, Hench et al. [1, 2] developed 45S5 comprising 45 mol% SiO_2_, 24.5 mol% Na_2_O, 24.5 mol% CaO, and 6 mol% P_2_O_5_, which has been commercially available as Bioglass^®^ since 1985 [3]. 45S5 bonds with bone and is clinically applied as a bone-grafting material in orthopedic surgery. In dentistry, 45S5 has been used for several dental applications such as resin composites [4–6], root canal sealers [7, 8], scaffolds [9], and toothpaste [10] owing to its promotion of hard tissue formation and integration with surrounding tissues.

When 45S5 contacts an aqueous environment (body fluids), it induces apatite layer formation on its surface owing to ion exchange [3]. The release of ions from 45S5, particularly sodium, calcium, and phosphate ions, increases the local pH of the surrounding environment. As regards 45S5, different compositions of silicate glasses in the system of SiO_2_-Na_2_O-CaO-P_2_O_5_ have been reported [11, 12], such as S53P4 (53 wt% SiO_2_, 23 wt% Na_2_O, 20 wt% CaO, and 4 wt% P_2_O_5_) [13], 58S (60 mol% SiO_2_, 36 mol% CaO, and 4 mol% P_2_O_5_) [14], and 13-93 (53 wt% SiO_2_, 6 wt% Na_2_O, 12 wt% K_2_O, 5 wt% MgO, 20 wt% CaO, and 4 wt% P_2_O_5_) [15]. These glasses were developed owing to their composition-dependent degradability, bone-forming ability, and anti-inflammatory properties.

An increase in pH owing to ions released from the aforementioned silicate glasses can reduce the number of bacteria sensitive to pH changes [2]. However, silicate glass is insufficient to effectively inhibit bacteria living in oral biofilms or tooth substrates [14,16]. Hence, several attempts have been made to provide silicate glasses with antibacterial effects by replacing certain components (Ca or Na) with metals such as silver (Ag), copper (Cu), zinc (Zn), or gallium (Ga), thereby exhibiting strong antibacterial properties. These metal-doped silicate glasses maintain their apatite-forming ability by releasing sodium, calcium, silicate, and phosphate ions, whereas the added metals confer antibacterial effects.

In recent years, there has been a growing interest in phosphate glass as a carrier for metal ion release. Phosphate glass is based on P_2_O_5_ as the glass network former containing CaO and/or Na_2_O as the network modifiers. The chemical composition of phosphate glasses can be easily manipulated. Hence, the release of ions incorporated into the glass can be regulated by the dissolution rate of the glass [17]. The duration, during which the glass exhibits antibacterial properties, can be adjusted by controlling the release of metal ions.

Dental caries has been recognized as an infectious disease induced by cariogenic bacteria. Developing restorative materials with antibacterial effects has become an important research topic in dental materials science. The control of bacteria around/beneath restorations could help eliminate the risks of further demineralization and cavitation, contributing to the prevention of secondary caries. Apical periodontitis is an infectious disease, and the treatment requires complete bacterial eradication inside filled root canals. However, it is challenging to achieve the complete elimination of bacterial infection in root canals using only mechanical instrumentation, irrigation, and medication. Therefore, endodontic filling materials capable of eliminating residual endodontic pathogens in root canals have been proposed as a possible solution to this problem. The application of metal-releasing glasses to dental materials has been attempted to address the growing need for antibacterial dental materials used for restorative/endodontic treatments. Further, a denture base with antimicrobial effects against fungi is also useful for the prevention of denture stomatitis.

In this review, metal-doped silicate and phosphate glasses with antibacterial activities are summarized. Furthermore, future strategies for the development of dental biomaterials that incorporate metal-doped glass and exhibit antibacterial effects are discussed.

## 2. Metal-doped glasses for conferring antibacterial activity

### 2.1. Silver-doped glass

Silver ions are widely recognized for their strong antibacterial properties and effectiveness against a wide range of bacteria. Silver ions and nanoparticles have demonstrated efficacy against different pathogenic bacteria, including *Staphylococcus aureus*, *Escherichia coli*, *Pseudomonas aeruginosa*, *Streptococcus mutans*, *Porphyromonas gingivalis*, *Enterococcus faecalis*, *Lactobacillus casei*, and *Actinomyces naeslundii* [18–23]. When silver is incorporated into glass, it releases silver ions that contribute to its antibacterial effect. Silver ions can disrupt bacterial cell membranes, thereby resulting in an increased permeability and leakage of cellular contents [24, 25]. They can also bind to sulfur-containing proteins and enzymes in bacteria, thereby interfering with their normal functions and inhibiting bacterial growth.

Over the past two decades, different Ag-doped silicate glasses have been developed [26–34](Table 1). Bellantone et al. [26] fabricated a silver-releasing glass comprising 76% silicon dioxide (SiO_2_), 19% calcium oxide (CaO), 2% phosphorus pentoxide (P_2_O_5_), and 3% silver oxide (Ag_2_O) in percentage of weight. The silver-doped glass in the range of 0.05 to 0.20 mg/mL of culture medium demonstrated bactericidal effects against *E. coli*, *P. aeruginosa*, and *S. aureus*. A complete killing effect was elicited within 24 h of incubation with 10 mg/mL silver-releasing glass. Phetnin et al. [29] investigated the antibacterial activity of Ag-containing mesoporous silicate glass. The agar diffusion test revealed that mesoporous glasses containing 1, 3, or 5 mol% Ag_2_O produced inhibition zones against *S. aureus* and *E. coli*. Hence, these mesoporous glasses have potential as drug-loading carriers with antibacterial properties.

**Table 1.** Metal-doped silicate glasses and their application to dental biomaterials.

Metal	Composition of glass	Application	Measured period of metal release	Reference
Silver	76 wt% SiO₂, 19 wt% CaO, 2 wt% P₂O₅, and 3 wt% Ag₂O	Glass only	168 h	[26]
			24 h	[30]
	60 mol% SiO₂, 34 mol% CaO, 4 mol% P₂O₅, and 2 mol% Ag₂O	Surgical sutures	N.A.	[27]
	70 mol% SiO₂, 29 mol% CaO, and 1 mol% Ag₂O	Glass only	172 h	[28]
	80 mol% SiO₂, (15 − x) mol% CaO, 5 mol% P₂O₅, and (x = 1, 3, or 5) mol% Ag₂O	Glass only	14 d	[29]
	60 wt% SiO₂, 6 wt% CaO, 3 wt% P₂O₅, 14 wt% Al₂O₅, 5 wt% Na₂O, 5 wt% K₂O, and 7 wt% Ag₂O	Pulp capping material (Addition of Ag-doped glass/chitosan hydrogel at 1:1 wt. ratio)	N.A.	[31]
		Resin composites (Addition of 10 wt% Ag-doped glass)	N.A.	[33]
	58 wt% SiO₂, 32 wt% CaO, 9 wt% P₂O₅, and 1 wt% Ag₂O	Orthodontic adhesive (Addition of 1 wt% Ag-doped glass)	N.A.	[34]
Copper	Cu/Ca/P/Si/ = 5/10/5/80 mol. Ratio	Glass only	7 d	[35]
	Cu/Ca/P/Si = x/(25 – x)/5/75 and x = 2 or 5	Eggshell membrane	7 d	[36]
	70 mol% SiO₂, 25 mol% CaO, and 5 mol% CuO	Glass only	7 d	[37]
	Cu/Ca/Si = 5:10:85 wt. ratio	Zinc phosphate cement (Addition of 2.5 wt% Cu-doped glass)	24 h	[38]
Zinc	58 wt% SiO₂, 28 wt% CaO, 9 wt% P₂O₅, and 5 wt% ZnO	Orthodontic adhesive (Addition of 5 wt% Zn-doped glass)	N.A.	[34]
	70 mol% SiO₂, (26-x) mol% CaO, 4 mol% P₂O₅, and (x = 3 or 5) mol% ZnO	Glass only	28 d	[42]
	42 mol% SiO₂, 5 mol% CaO, 53 mol% ZnO	Glass polyalkenoate cements (1 g Zn-doped glass, 0.36 g polyacrylic acid, and 0.55 mL water)	30 d	[43]
	42 mol% SiO₂, 5 mol% SrO, and 53 mol% ZnO			
	48 mol% SiO₂, 4 mol% SrO, 12 mol% CaO, and 36 mol% ZnO	Glass polyalkenoate cements (Zn-doped glass/polyacrylic acid and distilled water = 2:1.5 wt. ratio)	30 d	[44]
Zinc, Silver	56.04 mol% SiO₂, 32.76 mol% ZnO, 0.33 mol% Ag₂O, and 10.87 mol% Na₂O	Glass polyalkenoate cements (0.5 g Zn-doped glass, 0.2 g polyacrylic acid, and 0.25 mL distilled water)	30 d	[45]
Gallium	46.2 mol% SiO₂, 24.3 mol% Na₂O, 26.9 mol% CaO, 2.6 mol% P₂O₅, and (x = 1.0, 1.6, or 3.5) mol% Ga₂O₅	Glass only	30 d	[58]
	(80-x) mol% SiO₂, 15 mol% CaO, 5 mol% P₂O₅, and (x = 1, 2, or 3) mol% Ga₂O₅	Glass only	168 h	[59]

N.A.: Not applicable.

In dentistry, using silver-doped silicate glasses in pulp capping materials [31, 32], resin composites [33], and adhesives for orthodontic treatment [34] has been reported. Zhu et al. [31, 32] investigated the antibacterial activity of an injectable chitosan hydrogel pulp-capping material containing silver-doped silicate glass. When *S. mutans* and *L. casei* were incubated in the presence of the silver-doped glass/chitosan hydrogel at a 1:1 wt ratio, both bacteria were killed after incubation for 24 h. Lee et al. [34] examined the antibacterial effect of silver-doped glass comprising 58 wt% SiO_2_, 32 wt% CaO, 9 wt% P_2_O_5_, and 1 wt% Ag_2_O in a bisphenol-A diglycidyl ether dimethacrylate (Bis-GMA)/triethylene glycol dimethacrylate (TEGDMA)-based adhesive resin. The cured resin containing 1 wt. % Ag-doped glass inhibited the growth of *S. mutans* after incubation for 24 h.

### 2.2. Copper-doped glass

Copper is a vital mineral that is essential for biological processes. Most Cu in the human body is bound to enzyme prosthetic groups or proteins. Copper is nontoxic to human tissues and exhibits strong antibacterial activity.

The antibacterial properties of copper-doped silicate and phosphate glasses have already been reported, particularly in the last ten years [35–39](Table 1). Wu et al. [35] investigated the antibacterial activity of Cu-doped silicate glass (molar ratio of Cu/Ca/P/Si = 5/10/5/80) against *E. coli*. The copper-doped glass was mixed with *E. coli* suspension (3.5–4.0 × 10^4^ bacteria/mL), stored at 4 °C for 7 d, and incubated at 37 °C for 12 h. The copper ions released from the copper-doped glass demonstrated a killing effect against *E. coli*.

Choe et al. [38] developed zinc phosphate cement incorporating copper-doped silicate glass nanoparticles comprising 5 wt% Cu, 10 wt% Ca, and 85 wt% Si. When *E. faecalis* (10^6^ CFU/mL) was incubated in the presence of eluate from the zinc phosphate cements with/without copper-doped glass nanoparticles, the number of bacteria after incubation for 3 h was significantly decreased.

### 2.3. Zinc-doped glass

Silver- and copper-doped glasses have been extensively studied because of their ability to inhibit the growth of microorganisms. However, one of the major issues in incorporating metal-doped glasses into dental restorative materials is “discoloration”. When Ag or Cu ions are used at high concentrations or in certain formulations, they can cause noticeable discoloration of the material. This discoloration can be undesirable, particularly for restorative materials that must maintain their aesthetic appearance. To overcome this issue, researchers have explored the use of zinc, which may reduce discoloration (Table 1). The antibacterial action of Zn is still being studied, and its effects on bacteria vary depending on the specific species and strains involved. One potential mechanism by which zinc exhibits antibacterial activity against oral streptococci is through interference with bacterial glycolysis [40]. Glycolysis is the metabolic pathway that converts glucose to pyruvate and generates energy for cells. Zinc ions have been shown to inhibit enzymes involved in glycolysis, thereby disrupting energy production and metabolic processes in bacteria.

Several attempts have been made to incorporate zinc oxide (ZnO) into silicate [34, 42–44] or phosphate glasses [41, 47–49] (Tables 1 and 2). The addition of ZnO to these glass systems resulted in zinc-ion-releasing properties and antibacterial activity against a wide range of bacteria. Zn-doped silicate glasses have also been incorporated into glass polyalkenoate cements [43, 44]. Boyd et al. [43] fabricated two kinds of cements containing glass A (42 mol% SiO_2_, 5 mol% CaO, and 53 mol% ZnO) and glass B (42 mol% SiO_2_, 5 mol% SrO, and 53 mol% ZnO). They found that the release of Zn ion from both glasses exhibited antibacterial activity against *S. mutans* and *A. viscosus*, with the inhibition of growth greatest shortly after cement preparation and little or no inhibition measurable after immersion in water for 30 d. Coughlan et al. [45] reported that the addition of zinc and silver to silicate glass reduced bacterial viability when co-cultured with the elute from the glass for 2 h. Phosphate glasses are renowned for their substantial calcium and phosphate contents, which play a pivotal role in remineralization [46]. The dissolution rate of phosphate glasses can be easily adjusted by changing their chemical composition [17]. Lee *et al*. [47, 48] and Kim *et al*. [49] incorporated Zn-doped phosphate glasses into orthodontic acrylic resins/adhesives or resin composites and demonstrated their antimicrobial effects against oral microorganisms. However, these methods of incorporating Zn-doped silicate and phosphate glasses into restorative materials have only demonstrated short-term effects.

**Table 2.** Metal-doped phosphate glasses and their application to dental biomaterials.

Metal	Composition of glass	Application	Measured period of metal release	Reference
Copper	50 mol% P₂O₅, 30 mol% CaO, 10 mol% Na₂O, and 10 mol% CuO	Glass only	N.A.	[39]
Zinc	50 mol% P₂O₅, 25 mol% CaO, 20 mol% Na₂O, and 5 mol% ZnO	Glass only	N.A.	[41]
	42 mol% P₂O₅, 25.2 mol% CaO, 16.8 mol% Na₂O, and 16 mol% ZnO	Orthodontic acrylic resin (Addition of 3, 5, and 7 wt% Zn-doped glass)	24 h	[47]
		Resin composite (Addition of 1.9, 3.8, or 5.4 wt% Zn-doped glass)	24 h	[48]
	38 mol% P₂O₅, 22.8 mol% CaO, 15.2 mol% Na₂O, and 24 mol% ZnO	Orthodontic adhesive (Addition of 3, 6, or 9 wt% Zn-doped glass)	30 d	[49]
Gallium	45 mol% P₂O₅, 25 mol% MgO, 15 mol% CaO, 10 mol% Na₂O, and 5 mol% Ga₂O₅	Glass only	24 h	[60]
	45 mol% P₂O₅, 14 mol% CaO, 38 mol% Na₂O, and 3 mol% Ga₂O₅	Glass only	N.A.	[61]
Gallium, Silver	45 mol% P₂O₅, (x = 10, 11, or 12) mol% CaO, (47 − x) mol% Na₂O, 3 mol% Ga₂O₅, and 5 mol% Ag₂O	Glass only	48 h	[64]

N.A.: Not applicable.

### 2.4. Gallium-doped glass

Recently, extensive research has been conducted to examine the antimicrobial activity of Ga against different microorganisms [50–53]. Several studies have reported that Ga exhibits antimicrobial effects, particularly against Gram-negative bacteria, which are known for their increased resistance to numerous antibiotics [54, 55]. Gram-negative bacteria possess an outer membrane that acts as a protective barrier, thereby making it difficult for antimicrobial agents to penetrate and disrupt the cellular processes. The antimicrobial activity of Ga is owing to its ability to interfere with iron uptake by these organisms. Iron is essential for most bacteria, as it is required for enzymes that mediate numerous key processes such as DNA synthesis, electron transport, and oxidative stress defense. Gallium has a nearly identical ionic radius, coordination chemistry, and ionization potential to Fe(III) [56]. Given that several bacterial uptake systems are unable to distinguish between gallium and iron, gallium can substitute for iron and disrupt iron-dependent processes in bacterial metabolism [57]. By disrupting iron acquisition, Ga inhibits crucial processes within bacteria that rely on iron, such as the production of iron-containing enzymes and proteins necessary for growth and virulence. This disruption inhibits bacterial growth and increases the susceptibility of bacteria to the host immune system or other antimicrobial agents.

Several studies have reported the fabrication of gallium-doped silicate [58, 59] and phosphate [60, 61] glasses as carrier materials in terms of releasing gallium ions and have evaluated their antibacterial activities (Tables 1 and 2). Furthermore, gallium-doped glasses have been used as antibacterial materials against bone defects or skin incision infections [62, 63]. Valappil et al. [64] reported that gallium silver-doped phosphate glass inhibited the growth of *P. gingivalis* in biofilms in the constant-depth film fermentor model. Although the antibacterial effects against oral microorganisms have been previously investigated [61, 64], research is ongoing on the potential applications of gallium-releasing glasses as antimicrobial dental materials.

## 3. Future strategy toward development of antibacterial glasses

Numerous investigations on metal-doped antibacterial glasses have been reported. However, the following points highlight the complexities associated with them:

- Diminished antibacterial effect over time: Leaching of antibacterial metal ions from glass when exposed to saliva or other fluids can diminish the antibacterial effect over time. Continuous exposure to the oral environment may reduce the concentration of the released metal ions, thereby decreasing their long-term ability to combat bacteria.
- Disruption of oral microbial homeostasis: The oral cavity harbors a complex ecosystem of microorganisms, including beneficial bacteria, that contribute to oral health. Continuous and potent delivery of antimicrobial components may disturb this delicate balance, thereby resulting in the disruption of microbial homeostasis. This could potentially result in unintended consequences such as promoting the growth of antibiotic-resistant strains or affecting beneficial bacteria that help to maintain oral health. Moreover, the continuous release of metals into the oral environment may have significant implications on biological safety.

To overcome these limitations, the on-demand release of antimicrobial components in response to environmental changes is a promising approach. This approach minimizes the risk of disrupting oral microbial homeostasis and influences biosafety. Furthermore, the controlled release in response to environmental changes can prolong the duration of the antibacterial activity. An example of an on-demand release approach is glass, which releases metal ions in response to environmental acidity. This is beneficial in terms of providing dental materials with a controlled release ability to effectively supply antimicrobial ions when the acidogenic bacteria in dental plaques produce acids. BioUnion filler is a glass particle developed by GC corp., comprising SiO_2_-ZnO-CaO-F that is capable of releasing Zn^2+^, Ca^2+^, and F^-^ [65]. Liu *et al*. [66] reported that the release of Zn^2+^ from the BioUnion filler was accelerated under acidic conditions. This technology enables the on-demand release of antimicrobial components from dental materials. As a commercial material, a glass ionomer cement (GIC) containing BioUnion filler for root surface restoration (Caredyne^®^ Restore, GC Corp., Japan) is on the market. Liu *et al*. [67] reported that the acidity-induced release of Zn^2+^ from the GIC containing BioUnion filler effectively inhibited the growth and adherence of oral bacteria even after repeated exposure to acetic acid. Kohno et al. [68] assembled an original bioreactor and reported that the biofilm, similar to those formed on dental materials in the oral cavity, could be formed using a bacterial suspension prepared from human saliva and adding 0.2% sucrose solution. Using the established *in vitro* culture system, it was demonstrated that the GIC containing BioUnion filler effectively hindered oral biofilm formation on its surface compared to fluoride-releasing GIC and resin composites [68].

**Figure 1.**
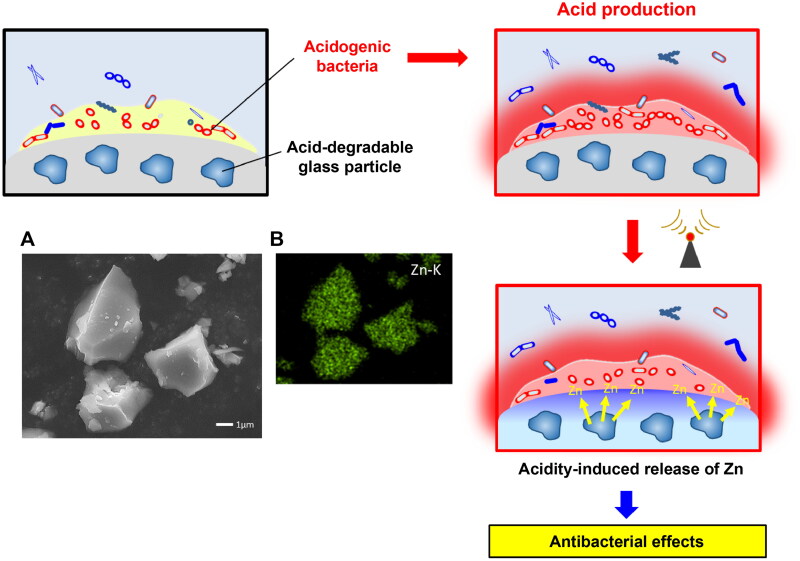
Acidity-responsive Zn-releasing glass particles. (A) Scanning electron microscope (SEM) and energy dispersive X-ray spectroscopy (EDS) mapping images of silicate-based glass particles containing 33 mol% Zn.

While the acidity-induced release of Zn^2+^ from BioUnion filler kills oral bacteria [66], the concentration of Zn^2+^ from restorative cement incorporating BioUnion filler is not considerably high enough to exhibit bactericidal effects against microorganisms in oral biofilm. Deng et al. [69] recently fabricated acidity-responsive silicate glasses containing high Zn concentrations (33–38 mol%; Figure 1). These glasses are modifications of the BioUnion filler that increase the Zn content and remove the Ca incorporated in the BioUnion filler. The glass particles with a greater Zn content demonstrated greater release concentrations of Zn^2+^ in acids. When *S. mutans* was anaerobically incubated in different pH media (5.1, 6.1, or 7.4), the number of viable *S. mutans* decreased in the presence of each particle with a decrease in the medium pH value. At pH 5.1, the number of viable cells in the presence of each particle was lower than the initial number of bacteria, thereby indicating that the glass particles exhibited bactericidal effects under acidic conditions. The glass particles that can release high concentrations of Zn^2+^ under acidic conditions would cause bactericidal effects in microorganisms in oral biofilms even when incorporated into the cements.

Dental caries is caused by acids from the glucose metabolism of specific bacteria in the oral cavity. One of the aims of conferring antibacterial activity to restorative or preventive materials is to suppress caries-related bacteria and inhibit the decrease in pH that occurs in dental plaques [70]. The aforementioned glass particles were capable of releasing Zn ions when the environmental pH decreased. Such an acidity-responsive ion-releasing technology that produces antibacterial effects as a result of environmental (pH or microbiota) changes is of significant interest. The future design of dental materials with ion-releasing glasses must involve smart behaviors to maintain the oral environment and safeguard human health.

Another promising approach involves recharging metal ions into materials that incorporate glass. Kohno et al. [71] investigated the release properties and recharging ability of GIC containing BioUnion filler using an *in vitro* saliva drop setting to simulate *in vivo* conditions of the oral cavity. The GIC incorporating the BioUnion filler was found to be rechargeable with Zn ions through the application of a tooth gel containing Zn. Following recharging, the effective concentration of Zn ions necessary to hinder the formation of a multispecies oral biofilm on the surface of the material was maintained. Such recharge properties are valuable for the long-term antimicrobial effects of dental materials as they can prevent the formation of multispecies oral biofilms on their surfaces.

Incorporating contact inhibition into dental materials is another smart approach to provide long-lasting antibacterial effects without releasing antimicrobial components [70]. Several studies have reported that silver nanoparticles or silver-containing materials (zirconium phosphate and silica gel) exhibit antibacterial activities not owing to the release of silver ions but rather owing to the activation of oxygen based on the catalytic action of silver [72, 73]. Silver generates reactive oxygen species (ROS) such as superoxide and hydroxyl radicals, which further contribute to bacterial cell damage [74, 75]. Hence, if dental materials containing metal-doped glass exhibit contact inhibition, they must exhibit long-lasting inhibitory effects against oral microorganisms on their surfaces.

In summary, various compositions of metal-doped silicate and phosphate glasses have been shown to enhance the antibacterial properties of dental materials. Employing approaches such as the on-demand release of ions and contact inhibition in dental materials are promising strategies to exhibit long-lasting antimicrobial effects and minimize disruption in the oral microbial ecosystem. The rechargeability of ions in materials containing metal-doped glasses can extend the duration of ion release and antibacterial activity. These innovative approaches are expected to affect oral health, thereby preventing the formation of biofilms and maintaining the balance of the oral microbiome.

## Data Availability

Data sharing is not applicable to this article as no new data were created or analyzed in this study.
